# Mechanobiology of the endothelium in vascular health and disease: in vitro shear stress models

**DOI:** 10.1007/s10557-022-07385-1

**Published:** 2022-10-03

**Authors:** Molly L. Jackson, Andrew Richard Bond, Sarah Jane George

**Affiliations:** Department of Translational Health Sciences, Bristol Medical School, University of Bristol, Bristol, BS2 8HW UK

**Keywords:** Atherosclerosis, Endothelium, Vascular disease, Vascular dysfunction

## Abstract

In recent years, there has been growing evidence that vascular pathologies arise in sites experiencing an altered haemodynamic environment. Fluid shear stress (FSS) is an important contributor to vascular homeostasis and regulates endothelial cell (EC) gene expression, morphology, and behaviour through specialised mechanosensitive signalling pathways. The presence of an altered FSS profile is a pathological characteristic of many vascular diseases, with the most established example being the preferential localisation of atherosclerotic plaque development. However, the precise haemodynamic contributions to other vascular pathologies including coronary artery vein graft failure remains poorly defined. To evaluate potential novel therapeutics for the treatment of vascular diseases via targeting EC behaviour, it is important to undertake in vitro experiments using appropriate culture conditions, particularly FSS. There are a wide range of in vitro models used to study the effect of FSS on the cultured endothelium, each with the ability to generate FSS flow profiles through which the investigator can control haemodynamic parameters including flow magnitude and directionality. An important consideration for selection of an appropriate model of FSS exposure is the FSS profile that the model can generate, in comparison to the physiological and pathophysiological haemodynamic environment of the vessel of interest. A resource bringing together the haemodynamic environment characteristic of atherosclerosis pathology and the flow profiles generated by in vitro methods of applying FSS would be beneficial to researchers when selecting the appropriate model for their research. Consequently, here we summarise the widely used methods of exposing cultured endothelium to FSS, the flow profile they generate and their advantages and limitations in investigating the pathological contribution of altered FSS to vascular disease and evaluating novel therapeutic targets for the treatment and prevention of vascular disease.

## Introduction

Endothelial cells (ECs) form the innermost lining of blood vessels and are essential for vascular homeostasis, with the quiescent endothelium maintaining an anti-coagulant, anti-inflammatory environment and regulating vascular tone and membrane permeability [1]. ECs are constantly exposed to a range of mechanical forces as a consequence of blood flow. Fluid shear stress (FSS,  ) is the tangential vector of the force exerted by blood flow and is defined as the frictional force of blood flow per unit area parallel to the vessel wall (dynes/cm^2^ or Pascals, Pa) [2]. FSS regulates vascular development, vascular homeostasis and the phenotype of ECs, contributing to vasoregulation following acute changes in blood flow and vessel wall remodelling in response to chronic haemodynamic alteration [3]. The translation of the detection and conversion of FSS as a mechanical stimulus to a biological response is known as mechanotransduction. ECs possess a range of mechanosensors including ion channels, receptor tyrosine kinases, G-protein coupled receptors and integrins that convert changes in the haemodynamic environment into the biochemical signals that modulate EC gene expression, behaviour, morphology and phenotype through specialised mechanosensitive signalling pathways [4]. The presence of an altered FSS profile is a pathological characteristic of many vascular diseases, with the most defined examples being the preferential localisation of atherosclerotic plaques in the coronary arteries at branches and bifurcations and in the development of vein graft failure (VGF) in the long saphenous vein following bypass grafting. Nevertheless, many in vitro experiments investigating novel drug therapies on the cultured endothelium use ECs in the absence of flow or exposed to an unphysiological FSS profile, therefore specific shear-dependent cellular responses can be missed and thereby limit clinical translation.

The physiological FSS profile varies depending on the vascular bed and size of the vessel and fluctuates temporally throughout the cardiac cycle [5]. As such, the haemodynamic environment variation between blood vessels produces vascular-bed-specific phenotypic heterogeneity with arterial and venous ECs being developed and maintained in different microenvironments containing distinct growth factor gradients, oxygen concentrations, adhesion molecule levels and shear stress levels that must be taken into account when selecting in vitro models and designing experimental conditions (summarised in Table 1) [6]. The FSS profile can be separated into distinct categories based on the flow directionality throughout the cardiac cycle (summarised in Table 2). Regions of the vessel free from vascular disease are most often associated with laminar and unidirectional FSS whereby blood flow acts as a constant tangential drag force on the luminal surface of ECs in a forward motion. The haemodynamic parameters thought to contribute to vascular pathologies have been reviewed extensively elsewhere from a biophysical perspective [7–11], and therefore only the most notable biological features relevant for selecting an in vitro model once the desired FSS parameters are determined are discussed here. The features of FSS thought to be altered in sites of vascular pathology include the presence of spatial and temporal gradients of FSS magnitude; the extent of the deviation of the FSS from the mean flow direction due to flow reversal throughout the cardiac cycle (oscillatory shear index, OSI: 0.0 unidirectional flow to 0.5 reversing flow with no mean forward motion [12]); the average FSS perpendicular to the temporal mean FSS vector distinguishing between uniaxial and multidirectional flow (transverse FSS [7, 13]); near-wall particle residence time calculated from the time-averaged FSS and OSI (relative residence time, RRT); and geometrical changes arising from vascular adaption and remodelling [14]. The flow profile is also defined by the Reynolds number (Re) which describes the stability of the flow by correlating the inertial forces to viscous forces and defines whether the flow is laminar or turbulent; Re = 4ρQ/πμD with ρ representing density, *D* vessel diameter, *μ* viscosity and *Q* flow rate [15]. Areas of uniform vessel geometry when exposed to the physiological range of magnitudes of laminar FSS have a low value Re (< 2000), OSI (< 0.15), transverse FSS and RRT [15]. Areas of recirculation and multidirectional flow associated with vascular pathology are found at vessel branches and bifurcations and have a low FSS magnitude (< 5 dynes/cm^2^) and a higher Re value (> 2000), which may become turbulent at areas of > 50% stenosis (Re > 4000) [16]. The flow at these atheroprone regions have also been associated with greater transverse FSS, OSI and RRT value [16]. Therefore, it is important that in vitro models of FSS have the capability to investigate these diverse parameters of FSS to better understand the contribution of the haemodynamic environment to the development and progression of vascular disease.

**Table 1:**
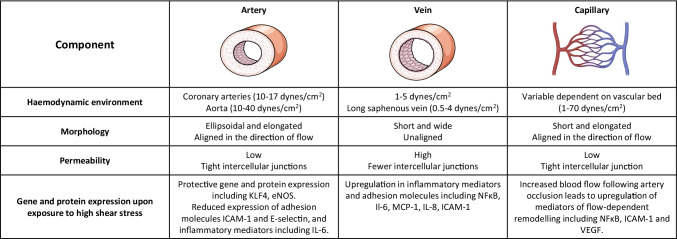
Phenotypic and functional heterogeneity of endothelial cells. ECs demonstrate vascular-bed-specific properties that alter their response to the exposure of FSS

**Table 2:**
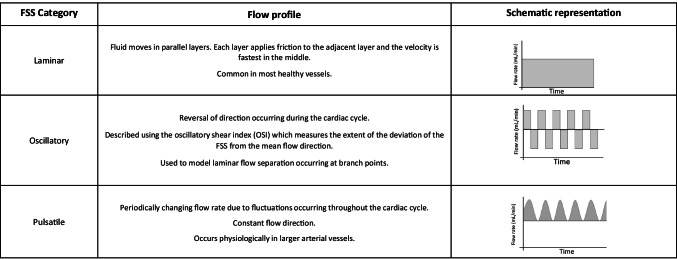
Shear stress categorisation based on directionality. The shear stress profile varies in different states of vascular health and disease with unidirectional and laminar FSS being associated with vascular health

## A brief summary of shear stress and vascular disease

### Atherosclerosis

Atherosclerosis is a chronic inflammatory condition of the artery that contributes to several vascular pathologies including coronary artery disease (CAD), myocardial infarction (MI) and ischaemic stroke [17]. Due to increasing socioeconomic change, increased lifespan and altered lifestyle associated risk factors, atherosclerosis is an increasing burden on both developing and developed countries [18]. Current pharmacological approaches in patients at risk of the development and progression of atherosclerosis focus on lowering the lipid burden and reducing inflammation [19]. In primary atherosclerosis, many studies report the observation of ‘atheroprotective’ high-magnitude laminar FSS (10–40 dynes/cm^2^) located at unbranched sections of the artery [20]. This atheroprotective FSS profile is associated with reduced endothelial permeability due to the presence of tight intercellular junctions between ECs which are also maintained in an anti-inflammatory and non-proliferative state [21]. The haemodynamic contribution to atherosclerotic plaque development and progression is evident due to the non-uniform distribution of atherosclerotic plaques which appear preferentially located at sites exposed to an ‘atheroprone’ FSS profile consisting of low-magnitude (< 5 dynes/cm^2^) complex secondary, recirculating flow patterns [3]. Such flow patterns are found at vessel branches and bifurcations which have periods of flow reversal throughout the cardiac cycle resulting in a change in the direction of deformation forces contributing to EC mechanotransduction [21]. These regions are associated with increased endothelial permeability, proliferation and leukocyte recruitment and adhesion which serves as a foundation for atherosclerotic plaque development [22]. Atherogenesis is also promoted in these regions due to reduced endothelial nitric oxide synthase production and vasodilation exacerbating further changes in the haemodynamic environment and resulting in adaptive vascular remodelling [23]. Early post-mortem studies first identified low-magnitude FSS (< 5 dynes/cm^2^) at sites of non-uniform vessel geometry which were observed to be prone to atherogenesis due to inflammatory EC activation and increased endothelial permeability and increased RRT allowing for the increased uptake of low-density lipoprotein (LDL) [12]. Further studies using laser Doppler anemometry on replicas of carotid bifurcations provided evidence that atherosclerosis occurs in areas of oscillatory flow [24]. These theories were subsequently combined into the definition of a pro-atherogenic FSS profile consisting of low-magnitude and oscillatory FSS. Over many decades, the low-magnitude oscillatory FSS theory was used as a basis for investigating the haemodynamic contribution to vascular pathology and for the development of mechanosensitive treatment approaches [25]. However, it is unclear which aspects of flow magnitude and directionality contribute to vascular pathology due to conflicting conclusions from computational fluid dynamics (CFD) studies which are dependent on accurate geometrical parameters and defined boundary conditions which vary between individuals [26]. For example, endothelial-to-mesenchymal transition, which has been implicated in the pathophysiology of atherosclerosis, was previously considered to arise from the exposure of ECs to oscillatory FSS [27]. Later studies, however, identified low-magnitude FSS components as contributing to this process, demonstrating that magnitude and directional effects of FSS must be considered both separately and in combination [28].

In recent years, there is also growing evidence that the multidirectional component of blood flow occurring at sites of branches and bifurcations contributes to atherosclerotic plaque development [7]. This multidirectional component of disturbed flow has been described using the transverse FSS metric which encompasses the average of components perpendicular to the mean FSS vector over the cardiac cycle ranging from 0 to the time averaged wall FSS value (Pa) [13]. A low transverse FSS indicates that the flow remains parallel to a single axis throughout the cardiac cycle, whereas a high transverse FSS value indicates large changes in the flow direction [7]. Indeed, in vivo studies using minipigs with intracoronary shear-modifying stents demonstrated an association between high transverse FSS, computed using frequency-domain optical coherence tomography, and a threefold increase in atherosclerotic plaque burden [29]. In vitro studies using a parallel plate flow chamber (12 dynes/cm^2^, 24 h) likewise demonstrated that flow direction relative to the morphological and cytoskeletal axis, rather than magnitude, is an important contributor to the endothelial response whereby protective production of nitric oxide was induced by parallel flow and pro-inflammatory NFκB activation identified in ECs exposed to perpendicular flow [30]. However, although the transverse FSS metric encompasses both flow directionality and magnitude, it does not discriminate between entirely forward or reversing unidirectional flow therefore it is important to consider this metric in combination with the time averaged FSS and OSI [13].

The haemodynamic environment may also be altered by age-related stiffening of the vascular wall due to reduced vascular compliance to accommodate mechanical forces [31]. As a result, it is likely that a range of FSS profiles, which are influenced by changes in the vessel geometry during ageing and disease progression, contribute to different stages of the development and progression of atherosclerosis [3]. Therefore, the use of in vitro models of FSS are important in identifying the haemodynamic contribution to the development of atherosclerosis to discover novel therapeutic targets.

### Vein graft failure

Coronary artery bypass grafting is the gold standard treatment for advanced coronary artery disease with autologous grafting of the long saphenous vein (LSV) being the most commonly used conduit to bypass coronary blockages and revascularise the ischaemic heart [32]. Despite the superior patency of its arterial counterparts, the LSV remains a commonly used conduit due to its ease of access due to its superficial location and sufficient length to bypass multiple diseased vessels [33]. Unfortunately, the LSV demonstrates 10–15% VGF within a year of surgery due to acute thrombotic development arising from technical mismanagement during surgery and from surgical procedures including high pressure distension in the prevention of venospasm and to detect the presence of leakage and the removal of the vasa vasorum during harvest [32]. Furthermore, despite improved surgical techniques including the no-touch method of vein harvest [34], up to 50% of grafts fail within 10 years following the development of vascular inflammation and intimal hyperplasia (IH) which serve as a foundation for the development of superimposed atherosclerosis and ultimately results in vessel occlusion [35]. As such, there is clear evidence that the altered haemodynamic environment is an important contributor to VGF [36]. Following graft implantation, venous endothelial cells (vECs) which are chronically adapted to low-magnitude FSS (1–5 dynes/cm^2^ [37]) become exposed to the high-magnitude pulsatile FSS of the arterial circulation (10–17 dynes/cm^2^). Upon exposure to the altered haemodynamic environment, vECs become activated and acquire a pro-inflammatory phenotype which in combination with intimal thickening results in the development of superimposed atherosclerosis which contributes to vessel stenosis and graft failure.

In addition to high-magnitude FSS, the development of vessel stenosis is preferentially located in the proximity of sites of anastomosis where non-uniformity in the vessel geometry produces a complex FSS profile with spatial and temporal gradients of magnitude and areas of recirculation and secondary flow [32]. Endothelial dysfunction arising from the alteration to the haemodynamic environment contributes to thrombosis in both early and late VGF due to the loss of anti-thrombotic factors including nitric oxide (NO) and prostacyclin (PGI_2_) and the generation and release of pro-thrombotic factors including thromboxane A2, fibrinogen and von Willebrand factor which result in shear-induced platelet activation, and adhesion [38]. Furthermore, at these sites of disturbed FSS, platelets release proliferative factors to the endothelium including platelet-derived growth factor [39]. The structural difference between the coronary artery and LSV is also thought to contribute to the development of VGF. The LSV consists of variable lumenal diameter, a narrower medial layer composed of a discontinuous and irregular elastic lamina, fewer vascular smooth muscle cells (VSMCs) in the tunica media, an adventitia composed of highly aligned longitudinal collagen fibres [40] and valves. The thinner and less elastic medial layer, in conjunction with the collagen structure, reduces the ability of the LSV to passively expand in response to the high arterial pressure [40]. As a result, the variability of the lumen and the compliance mismatch in the vein graft further exacerbates changes to the haemodynamic environment to which the ECs within the graft are exposed [41]. Pharmacological interventions have been widely implemented for the prevention of early VGF. The prescription of aspirin as an anti-platelet agent following surgery has shown a great reduction in the risk of acute thrombotic events. Aspirin may also be prescribed in conjunction with ticagrelor or clopidogrel as a dual anti-platelet therapy which has demonstrated a reduced 1-year incidence of sudden cardiac death, MI or stroke [42, 43]. Additionally, vessel occlusion due to late stage VGF occurs in 50–60% LSV grafts within 10 years and therefore interventions targeting inflammation and IH are required to ensure long-term quality of life for CAD patients.

## In vitro methods of applying shear stress

Due to the profound impact of FSS on EC function and behaviour in health and disease, effective in vitro FSS models to produce controlled flow parameters are required to increase the understanding of the haemodynamic contribution to physiological and pathophysiological vascular function, to identify novel therapeutic targets and to validate and further develop novel treatments for vascular disease [20]. Understanding the haemodynamic contribution to vascular disease also presents a unique opportunity in the growing field of nanoscience whereby it has been suggested that the distinct FSS profile in stenosed vessels provides an opportunity for the development of mechanosensitive drug delivery systems and targeted drug delivery (as reviewed [44]) which is currently limited by a lack of cardiovascular disease-specific therapeutic targets. In vivo animal vascular studies have made an important contribution to the understanding of vascular mechanobiology [45]. However, the complexity of vessel geometry, the varying conclusions from CFD studies and the resolution limitations of technology to measure FSS in vivo has resulted in the need for in vitro models covering the wide range of FSS profiles. In vitro models of FSS are also important from an ethical standpoint, being commonly used to validate potential therapeutic targets of interest prior to preclinical animal studies. There are a wide range of methods used to study the effect of FSS on cultured ECs; therefore, an important consideration to select an appropriate model is the FSS profile that the model generates in comparison to the haemodynamic environment of the vascular physiology and pathophysiology being investigated. The computation of the fluid dynamics involved in producing the FSS profile described in the following models have been reviewed in detail elsewhere [46–49], and only the unique benefits and limitations for using the mentioned models from a biological perspective are discussed here and summarised in Table 3.

**Table 3:** Summary of the benefits and limitations of in vitro methods of exposing endothelial cells to fluid shear stress. The parameters affecting FSS profile and the benefits and limitations of use vary between models and must be taken into consideration by the investigator based on the experimental design

Features of model	Cone and plate device	Orbital shaker	Parallel plate flow chamber	Ibidi air pressure pump	Microfluidic devices
FSS magnitude range	Commonly used at 0–50 dynes/cm^2^	Commonly used at 0–50 dynes/cm^2^	0.5–40 dynes/cm^2^ commonly used	0–245 dynes/cm^2^ dependent on channel slide and tubing volume	0.7–130 dynes/cm^2^
FSS directionality range	Laminar, oscillatory, pulsatile, uniaxial	Laminar, oscillatory, pulsatile, uniaxial, multiaxial	Laminar, oscillatory, pulsatile, uniaxial, multiaxial	Laminar, oscillatory, pulsatile, uniaxial	Laminar, pulsatile, uniaxial, oscillatory
Factors affecting FSS profile	Cone rotation velocity and height, rotation radius and the fluid viscosity	Fluid depth and viscosity, well radius, orbital radius and angular velocity	The height and width of the fluid path, flow rate driven by the pump and chamber geometry	Air pressure (mbar), flow rate, tubing size, channel slide volume and channel slide geometry	Chamber geometry and volume and flow rate
Experimental applications	RNA extraction, protein lysate collection, fixed cell imaging, detection of cell signalling and factors released into conditioned medium	RNA extraction, protein lysate collection, fixed and live cell imaging, detection of cell signalling and factors released into conditioned medium, permeability assays, co-culture, wound healing, leukocyte transmigration	RNA extraction, protein lysate collection, fixed and live cell imaging, detection of cell signalling and factors released into conditioned medium, permeability assays, co-culture, wound healing, leukocyte transmigration	RNA extraction, protein lysate collection, fixed and live cell imaging, detection of cell signalling and factors released into conditioned medium, permeability assays, co-culture, wound healing, leukocyte transmigration	Endpoint applications limited by low cell number. Can be used in live and fixed cell imaging, co-culture, immune cell interactions
Working volume	10 mL medium for 10 cm cell dish	< 3 mL	Dependent on tubing circuit used, commonly up to 30 mL	2.5–12 mL dependent on tubing circuit used	Low, dependent on tubing. Chamber width commonly up to 200 μm
Time	Acute and chronic	Acute and chronic	Acute and chronic	Acute and chronic	Acute and chronic
Cost	Low	Low	High	High	High
Set-up	Consists of ECs seeded into a medium-filled dish where a cone driven by a connected motor is placed in close proximity	Simple, requires tissue culture plate and orbital shaker	Requires pump or syringe to drive flow of media through a circuit, commonly using a glass media bottle with inlet and outlet tubing	Requires commercially available specialist equipment	Requires pump to drive flow of media and chambers designed to achieve desired FSS profile
Throughput	Low, however variations using multiple cones connected to a single motor allow for increased output	High, multiple plates can be run at the same time	Low, although configurations using multiple slides can increase throughput	Low, although configurations using chamber slides connected in series can increase throughput	Multiple channel configurations allow for high throughput covering a range of FSS profiles

## Cone and plate

**Fig. 1:**
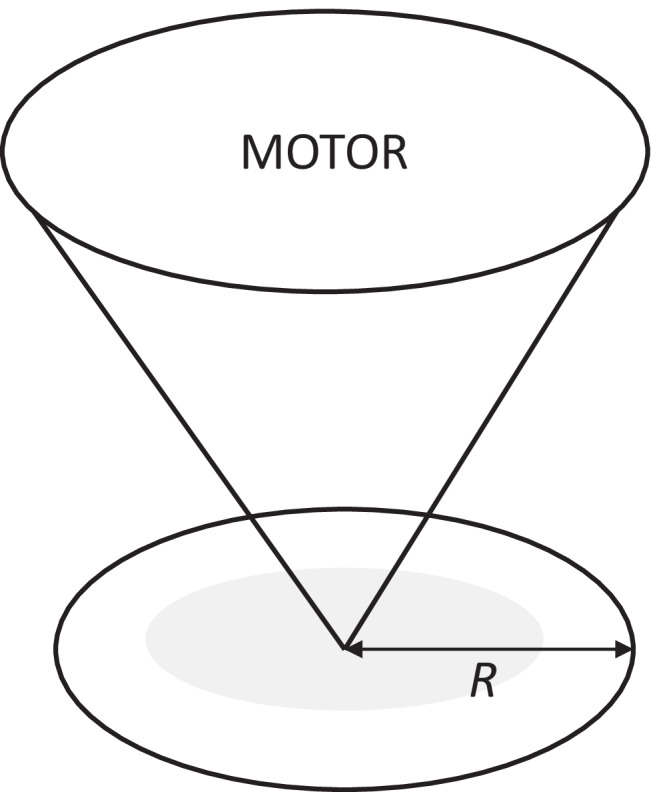
Schematic representation of the cone and plate device. The CPD consists of an endothelial monolayer cultured in a dish (grey) with a cone placed in close proximity controlled by a programmable motor to drive its rotation and generate a complex waveform. The rotation radius (R) and speed may be modified in line with numerical simulations and computational fluid dynamics to generate the required magnitude and directionality of the flow profile

The cone and plate device (CPD) is a widely used in vitro FSS model that has successfully been used to characterise key aspects of mechanobiology of ECs in the development and treatment of atherosclerosis. Developed in the 1930s, the CPD has been used to investigate mechanobiological processes for many decades [50]. The CPD consists of ECs seeded into a medium-filled dish where a cone is placed in close proximity (Fig. 1). The rotation of the cone, driven by a connected motor, creates a complex waveform with the ability to generate dynamic variation in FSS directionality and magnitude relevant to the physiological and pathophysiological range for ECs commonly used to investigate EC mechanobiology (0–50 dynes/cm^2^) [51]. The magnitude of FSS can be varied by modulation of the cone rotation velocity and height, the rotation radius and the fluid viscosity [52, 53]. The CPD also allows for long-term exposure to FSS; therefore, this model is well-suited for studying both the acute and chronic endothelial response (seconds–weeks) [52]. The system is limited by its low throughput in exposing ECs in a single dish to FSS; however, variations using multiple cones connected to a single motor allow for increased output [54]. Using this model, it is possible to generate FSS of varying magnitudes and directionality. The quantification and analysis of the magnitude and directionality of the FSS profile is determined by the equation ω = τ ∗ α/μ where ω is cone angular velocity (rad/s); τ, FSS (Pa); α, cone angle (rad); μ, dynamic viscosity (Pa.s) [52]. In addition, numerical simulations and CFD are used to predict FSS as a function of time based on the rotation speed of the cone to generate variable FSS profiles (computational analysis out of scope, further information found at [51, 52]). This flow profile, despite the ability to change the direction of flow for use in modelling oscillatory and pulsatile FSS, is still largely considered uniaxial which may limit its modelling of vascular pathology [7, 53, 55]. The CPD has contributed to the understanding of vascular mechanobiology in atherosclerosis under physiological conditions in a range of studies. For example, the CPD was used to identify LIM zinc finger domain containing 2 (LIMS2) as a mechanosensitive gene which is downregulated by low-magnitude oscillatory FSS (± 5 dynes/cm^2^ vs 20 dynes/cm^2^, 24 h) characteristic of the FSS profile of the branches and bifurcations at which atherosclerotic plaques are preferentially localised [56]. The downregulation of LIMS2 was associated with an upregulation of inflammatory markers and therefore may provide insight into the endothelial inflammation that serves as a foundation for atherogenesis and provide a therapeutic target for the flow-dependent development of vascular disease [56]. Furthermore, using the CPD to expose the cultured endothelium to laminar arterial FSS (20 dynes/cm^2^, 12–48 h) contributed to the elucidation of the atheroprotective role of NOTCH1 as a mechanosensor in adult arteries with ECs demonstrating aligned morphology, suppressed proliferation and an anti-inflammatory phenotype characteristic of atheroprotective areas of the vessel, which was lost in siRNA knockdown ECs [57]. In this study, the CPD was used for RNA and protein lysis experiments due to the FSS exposure to a large number of cells, which is limited in alternative in vitro FSS models. The CPD has also been used to investigate the effect of cardiovascular risk factors and the response to FSS. In particular, studies using the CPD to induce laminar low FSS (5 dynes/cm^2^), high FSS (20 dynes/cm^2^) as an exercise mimetic in comparison to static ECs demonstrated differences in levels of oxidative stress and inflammation which were attenuated by the application of laminar FSS [58]. This study demonstrated further evidence for lifestyle changes as an important nonpharmacological therapy for those at increased risk of cardiovascular disease.

## Orbital shaker

**Fig. 2:**
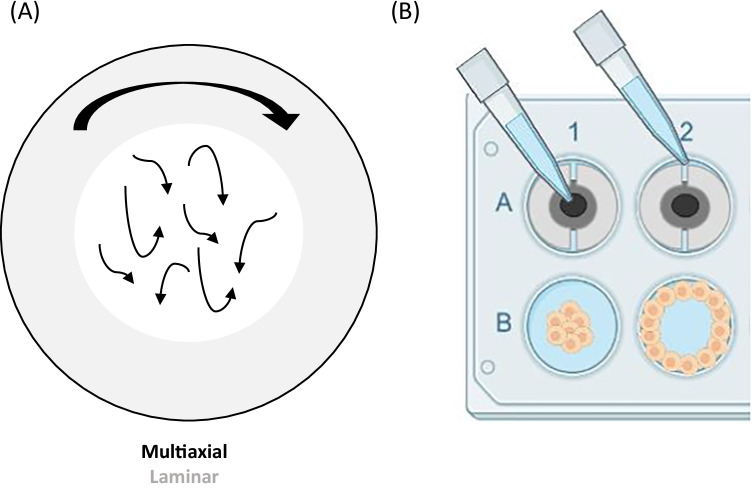
The orbital shaker generates distinct flow profiles using an orbital rotation in the horizontal plane. The orbital shaker mechanism of including shear stress requires an orbital shaker and a monolayer of cultured ECs. The orbital radius, fluid depth and viscosity and the angular radius can modify the magnitude of the shear stress profile. **A** Cells are exposed to low-magnitude multiaxial FSS in the centre of the well (white) and the cells at the edge are exposed the laminar shear stress (grey) with the magnitude depending on controlled parameters. **B** To prevent the effects from the release of factors from cells exposed to contrasting shear stress profiles, a segmentation approach can be used to seed cells specifically on the centre or edge of the well

The orbital shaker (OS) has long been used as a tool for sample agitation and mixing, and in recent years has been increasingly used to generate multidirectional FSS. The OS consists of a motorised platform, on which cultured ECs are placed, rotating in the horizontal plane around a defined orbital radius. The fluid depth and viscosity, well radius, orbital radius and angular velocity can be controlled to set a defined FSS output with each parameter modulating both FSS magnitude and directionality [59–61]. The use of this system benefits from a simple and cost-effective set up, high throughput, ability to permit both acute and chronic exposure to FSS and with low media volumes (< 3 mL) which is beneficial for the collection of conditioned media and maintaining a low-cost for the delivery of pharmacological reagents [62]. A CFD approach has been used to quantify and characterise the FSS profile which is divided into two distinct regions consisting of low-magnitude (< 5 dynes/cm^2^) multidirectional FSS in the centre of the well and unidirectional FSS around the edge (> 10 dynes/cm^2^) [63]. EC media used to generate the waveform is a Newtonian fluid and as such has a constant velocity and viscosity; however, these parameters can be customised to generate a specific FSS profile (as reviewed in [64]). It has been well-established that the ECs seeded in the centre and edge of the well are exposed to distinct FSS profiles which may result in the release factors that may influence the behaviour of cells located at different regions of the well (Fig. 2A) [65]. To overcome this limitation, segmentation approaches have been developed to restrict the adherence and growth of cells to regions with the desired FSS profile (Fig. 2B) [65]. The OS is limited by the requirement of the quantification of FSS magnitude to be determined numerically using comparisons based on CFD and simulations of the travelling waveform. Furthermore, it must be considered that as a travelling waveform, the FSS generated by the OS may not be fully uniform and the ECs are exposed to a range of FSS magnitudes instead of a defined value (reviewed in more detail [66]). Reassuringly, values derived from CFD simulations have been validated experimentally using optical Doppler velocimetry and particle image velocimetry demonstrate a general agreement between the experimental and numerical approaches [59, 61, 63]. Taking these considerations into account, this model has been widely used to investigate the contribution of atheroprone and atheroprotective FSS to endothelial function. For example, the OS was used to investigate protective factors released from ECs exposed to the putative atheroprotective flow (> 10 dynes/cm^2^, unidirectional, 72 h), identifying increased follistatin-like 1 protein levels in EC-conditioned media, which was associated with a reduction in transcytosis and inflammation indicative of the EC phenotype found in regions exposed to laminar uniform FSS [62]. This study indicated that the multidirectional component of FSS can promote EC inflammation and increased membrane permeability atheroprone regions of the vessel. Furthermore, the therapeutic application of follistatin like-1 protein or a compound acting via the same mechanism may have therapeutic benefit in reducing atherosclerotic plaque burden in atheroprone regions. Although beneficial for investigating arterial physiology and pathophysiology, the OS system is also limited by the development of numerical simulations and experimental quantifications based on generating arterial FSS (> 10 dynes/cm^2^) to compare the putative atheroprotective and atheroprone flow profiles observed in the development of primary atherosclerosis. As a result, the conditions required to generate low-magnitude unidirectional FSS (< 5 dynes/cm^2^) for the investigation of venous pathology including VGF and comparisons of vascular-bed-specific responses remain largely uncharacterised. Defining the parameters required to induce a venous FSS profile would further increase the ability to use this system to investigate additional aspects of vascular pathology.

## Pump systems

**Fig. 3:**
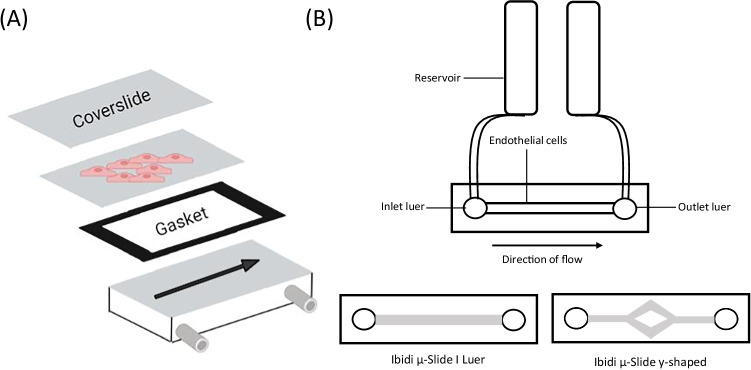
The use of pump systems to generate shear stress profiles with varying magnitude and directionality. **A** Representation of the parallel plate flow chamber system using a peristaltic or syringe pump to drive media across cells seeded on a coverslip or slide. **B** The Ibidi system uses an air pressure-driven pump to drive laminar unidirectional or oscillating flow across cells seeded in a channel slide with built-in coverslip

Another commonly used method of exposing the cultured endothelium to FSS is the parallel plate flow chamber (PPFC) [67]. The PPFC system uses a pump to drive media through a chamber containing ECs cultured on a glass coverslip or slide (Fig. 3A) [68]. The FSS magnitude is determined by a silicone gasket that establishes the height and width of the fluid path and the flow rate driven by the pump, with the FSS profile calculated using the equation which applies to Newtonian, incompressible flow: *τ* = 6μǪ/wh^2^ with *τ* representing FSS; *μ*, viscosity; *Ǫ*, flow rate; *w*, width and *h*, height. This set-up allows the generation of laminar FSS of varying magnitudes (0.5–40 dynes/cm^2^ commonly used to model the range of vessels throughout the circulatory system) and can be used as a closed circulation or one-way perfusion system depending on the experimental application. The introduction of tapering devices or tiered channel inserts to produce a step-flow design has allowed the generation of a FSS gradient stimulus and multidirectional flow to be applied (PPFC configurations as reviewed in more detail, [69]). The simple set-up and calculation of the FSS profile to which the ECs are exposed has resulted in this system being widely used to investigate the cultured endothelium [68]. However, previous studies demonstrate that the generated FSS profile contain areas of non-uniformity including at the walls and reservoirs of in vitro FSS models, including the PPFC, whereby FSS can be up to 80% of that calculated from the given flow rate depending on the chamber design [70]. These areas of non-uniformity may result in the release of factors from ECs exposed to different FSS profiles. Numerical solutions have been used to identify regions of the coverslip in which the FSS profile is in close proximity to that calculated from the given flow rate and fluid path [70]. As a result cells must be seeded at an appropriate entrance and exit length to be exposed to laminar flow (as reviewed in more detail 71]). The PPFC system has been widely used to characterise the contribution of haemodynamic factors to vascular pathology, with NFκB identified as a regulator of acute EC inflammation in the LSV when exposed to the acute arterial high FSS (12 dynes/cm^2^ vs 0.5 dynes/cm^2^, < 6 h) vECs experience following vein graft implantation in VGF [72]. This rapid pro-inflammatory activation of vECs exposed to arterial FSS demonstrates a role of changes to FSS magnitude in VGF and suggests that the inhibition of pro-inflammatory pathways such as NFκB prior to graft implantation may be beneficial in preventing this response [72]. As such, the use of NFκB inhibitors such as BMS-06 demonstrated reduced atherosclerotic plaque burden in Apolipoprotein E null mice [73]. The PPFC has also been utilised in identifying therapeutically relevant targets which have shown success in clinical trials. For example, the PPFC (3–6 dynes/cm^2^) was used to demonstrate a pathological increase in the expression of proprotein convertase subtilisin/kexin type 9 (PCSK9) in ECs and VSMCs when exposed to atheroprone low-magnitude FSS [74]. PCSK9 contributes to the degradation of low-density lipoprotein (LDL) receptors; therefore, its inhibition has emerged as a possible therapeutic to reduce hyperlipidaemia and cardiac diseases including atherosclerosis [75]. Indeed, clinical trials using PCSK9 inhibitors have subsequently demonstrated a reduction in low-density lipoprotein levels (LDL) by enhancing LDL uptake and clearance in the liver and an associated reduction in cardiac events [74, 75]. It is therefore possible that PCSK9 inhibitors have additional beneficial effects on blood vessel ECs combating deleterious effects of FSS as well as beneficial effects within the liver. The PPFC has also been used to investigate the mechanism of action and relationship to FSS of pre-existing cardiovascular drugs. In particular, the PPFC used to induce high unidirectional laminar shear stress characteristic of the arterial circulation (12 dynes/cm^2^) demonstrated that a reduced concentration of commonly used cardiovascular drug, atorvastatin, was required to protect against reactive oxygen species (ROS)-mediated damage demonstrating the synergistic effect of drugs and the haemodynamic environment [76]. The use of this system may be limited for certain experimental applications due to the requirement of a media reservoir with a volume large enough to flow through the circuit, commonly using a glass media bottle with inlet and outlet tubing to achieve this, limiting the analysis of shear-dependent EC functions and interactions with surrounding cells [77, 78]. The pump chosen may also have implications on the study output. Mechanical stress induced by peristaltic pumps may result in the release of factors due to non-specific activation of circulating cells, for example in monocytes used for flow adhesion assays [79]. To overcome this limitation, a syringe pump may also be used as a flow generator, consisting of a rotating piston that presses the plunger at a desired flow rate to drive media over cultured ECs for short-term experiments usually requiring one-way perfusion, although longer term exposure and pulsatile FSS generation is possible using configurations with multiple syringes [80, 81].

More recently, Ibidi have developed an air pressure-driven pump system that exposes ECs seeded into a channel slide with a built-in coverslip to laminar FSS (Fig. 3B) [57, 82]. Software allows the input of a FSS magnitude in dynes/cm^2^ with the required pressure (mbar) and flow rate (mL/min) automatically calculated based on tubing size and channel slide volume. As a result, as a readily available commercial device, this system produces a validated and well-characterised uniform FSS profile. Using a commercially available device is beneficial for the comparison of results between studies and for comparisons made in meta-analyses. The air pressure pump generates a range of FSS profiles, including pulsatile flow associated with the physiological environment of the arterial circulation and the oscillatory flow that has been identified as a component of the altered haemodynamic environment in several vascular pathologies. Nevertheless, this oscillatory flow is largely uniaxial and multiaxial FSS direction-specific pathological effects may be missed [7]. Although the flow profile generated is largely uniaxial, the introduction of Y-shaped channel slides enables small regions of multiaxial flow at the branch point to be generated. However, it is currently not possible to isolate cells specifically from this small region and as such this cannot be used as a model of multiaxial flow. Instead, this system requires custom-made chambers to achieve required parameters required for a specific experimental study design outside of commercially available products [69]. Furthermore, the limited size of the FSS chambers may provide limitations in extracting material for experiments such as Western blots, although configurations using multiple chambers connected in series may be utilised to increase cell numbers to be sufficient for lysate collection. Also, as in the PPFC, the chamber geometry produces a different FSS magnitude at the edge of the chamber and the areas in proximity to the inlet and outlet luers that must be taken into account [83]. The application of unidirectional, pulsatile and oscillatory FSS has contributed to the understanding of early atherogenesis. For example, this system was used to elucidate the FSS directionality-dependent role of CCN1, which is expressed in advanced atherosclerotic plaques, in NFκB activation and the development of an atheroprone EC phenotype modulation by comparing ECs exposed to unidirectional high-magnitude laminar FSS (12 dynes/cm^2^, 24 h) and oscillatory FSS conditions (± 5 dynes/cm^2^, 24 h) [84]. The use of the Ibidi system to generate unidirectional laminar FSS also elucidated the direction-dependent mechanosensitive modulation of Yes-associated protein (YAP) activation. ECs were exposed to atheroprotective laminar FSS (12 dynes/cm^2^) which promoted YAP autophagy and resulted in a downregulation of pro-inflammatory gene expression and a reduction in the risk of atherosclerotic plaque development [85]. In contrast, YAP activation was observed following exposure to oscillatory disturbed flow (± 0.5 dynes/cm^2^) providing further evidence that FSS directionality contributes to the atheroprone phenotype of ECs located at areas vulnerable to atherogenesis. Indeed, further studies demonstrated that the application of the YAP inhibitor verteporfin, currently used in the treatment of macular degeneration, demonstrated increased apoptosis of macrophages in the atherosclerotic plaque and reduced VSMC content [86]. Furthermore, approved drugs such as statins inhibit the YAP/TAZ pathway which is thought to reduce the inflammation and proliferation resulting from exposure to disturbed flow [87]. The use of an air pressure pump also limits non-specific activation of circulating cells in flow adhesion assays by reducing mechanical stress and the enclosed perfusion system allows the investigation of cell signalling between ECs in the channel slide and between ECs and circulating cells [88]. Due to the wide range of FSS profiles generated and the scope for multiple different assays including circulating cell interaction, the Ibidi system has proved beneficial in being used as a screening method prior to use in animal studies. For example, CX_3_CR1 has previously been identified as a target for the prevention of inflammation and monocyte adhesion in cardiovascular disease and its antagonist AZ1220 was developed into a drug-eluting stent [89]. Prior to deployment in a large animal model, the efficacy of AZ1220 was validated using the Ibidi pump system (at 1 dyn/cm^2^) to carry out monocyte perfusion experiments [89]. It must be considered however that this system is limited by the high cost of the specialised equipment and low throughput. In addition, the large volume of the media reservoir requires the concentration of conditioned medium for the detection of released factors into conditioned media and results in a high cost of pharmacological reagents.

## Microfluidic devices

**Fig. 4:**
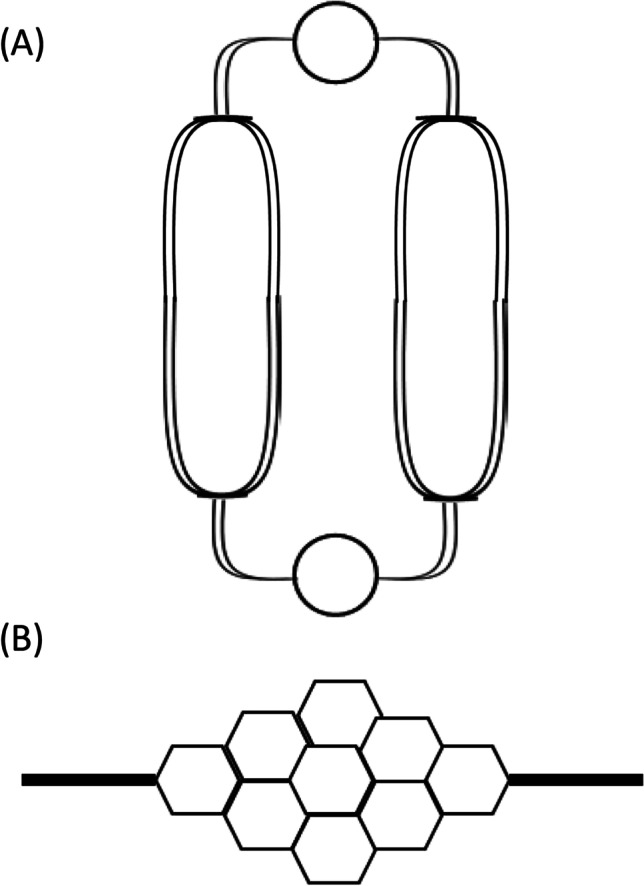
The use of microfluidic devices to apply FSS to culture ECs. **A** The use of multiple channels in a single chip allow the generation of multiple FSS magnitudes. **B** Microfluidic channel configurations can be modified to produce varying FSS profiles along the channel

The CPD, OS and pump systems are classified as macro-scale techniques to apply FSS to the cultured endothelium. For a greater level of control over the cellular microenvironment utilising a small number of cells, the investigator may choose to use microfluidic devices (Fig. 4). The use of multiple channels in a single chip allow the generation of multiple FSS magnitudes (0.7–130 dynes/cm^2^) and are able to generate laminar and pulsatile FSS profiles characteristic of the physiological venous and arterial circulation, respectively [90]. Microfluidic devices also provide useful tools to investigate vascular pathology, as they can produce a disturbed oscillating waveform (± 4–7 dynes/cm^2^) and FSS magnitude gradients with the use of tapering devices allowing the investigator to customise the FSS magnitude and directionality in accordance to the FSS profile vascular disease modelled [91]. These devices also require a small media volume reducing the volume required of reagents for pharmacological manipulation of ECs. Due to their size, microfluidic devices accommodate low EC numbers which may be beneficial for investigating the regional heterogeneity of the microscopic FSS force acting on individual cells throughout the endothelial monolayer which may be relevant to the focalised nature of atherosclerotic plaque development in primary atherosclerosis and VGF [92]. Microfluidic devices have contributed to the development of novel therapeutic approaches to specially target areas of vascular disease. For example, microfluidic devices with a tapered design to simulate areas of vessel stenosis have been used to validate the distribution of nanoparticle delivery with the aim to develop mechanosensitive treatment therapies (reviewed in more detail [93]). Likewise, the use of microfluidic devices is becoming an established approach to improve the in vitro to in vivo translation of novel drug delivery vehicles due to the increased relevance of the presence of FSS to physiological and pathophysiological conditions in vivo and the ability to control the flow rate and observe the interactions between the vehicles and cells [94].

## Limitations

There remain many challenges in the in vitro modelling of biological mechanical forces. There is limited ability to accurately measure the boundary conditions and the exact FSS forces acting on the ECs due to the limited resolutions of current detection approaches [26]. Furthermore, the difference in Newtonian and non-Newtonian fluid dynamics must be taken into account with a majority of in vitro FSS models using media with a constant viscosity and density which contrasts to the dynamic viscosity of blood as a non-Newtonian fluid [8]. The endothelial response to flow may also show patient variation with the possibility of mutations in mechanosensitive genes and the presence of co-morbidities and cardiovascular risk factors such as diabetes, hypertension and hyperlipidaemia which may produce a defective flow response [95]. As a result, the source of primary cells for the in vitro investigation of the haemodynamic environment must be carefully considered relative to the vascular pathology, and possible associated co-morbidities, being studied. Furthermore, as mentioned previously, the physiological and pathological FSS profile to which ECs are exposed to varies with and is dependent on the vascular bed from which they originate [6]. As a result, the type of primary ECs must also be considered. For example, despite arterial and venous ECs developing in and being maintained in different microenvironments containing distinct growth factor gradients, oxygen concentrations, adhesion molecule levels and shear stress levels, human umbilical vein ECs (HUVECs) are commonly used to model arterial atheroprotective and atheroprone FSS due to their low cost and availability [96]. However, due to this being an unphysiological FSS profile, specific shear-dependent cellular responses can be missed. As a result, the lineage of the ECs used should be considered as should the EC species, due to species difference in the haemodynamic environment, and vascular bed location for example using specialist cells including human lung microvascular cells (HMVEC) or glomerular endothelial cells (GenC) depending on the system being investigated (as reviewed in more detail [97, 98]). A physiologically relevant timecourse of FSS application must also be considered as acute and chronic application of FSS have been shown to induce opposing effects on the endothelium [99]. In vivo, ECs are exposed to a range of mechanical forces contributing to mechanotransduction, including cyclic strain and the circumferential stretch of the vessel wall which remains challenging to model in macro-scale in vitro FSS models due to limitations of the cultured endothelium including cell adhesion to a hard, inflexible surface lacking aspects of vessel geometry. However, the use of hydrogels and flexible substrates in the in vitro models covered in this review also allows for the generation of stiffness gradients which may be used to investigate aspects vascular disease including the age-relating vascular stiffening [31, 40]. Furthermore, the ability to culture cells on hydrogels also allows for the modelling of a more physiologically relevant microenvironment and allows for the investigation of a combination of additional haemodynamic force stimuli such as mechanical strain (beyond the scope of the review) [100].

## Conclusions

Shear stress is an important contributor to vascular physiology but its role in vascular pathogenesis remains to be fully clarified. There are a wide range of in vitro methods of applying shear stress to cultured ECs producing a range of shear stress profiles. There has previously been an emphasis on using these models to compare ‘atheroprotective’ and ‘atheroprone’ shear stress profiles in primary atherosclerosis. Importantly, with growing understanding of the contribution of both magnitude and directional-dependent changes in the haemodynamic environment to vascular pathologies these models may be translated for wider applications to investigate vascular function. There is no single perfect model of applying shear stress in vitro however, by characterising the FSS profiles generated by in vitro models and pairing them to the FSS profiles identified in vascular disease, these mechanisms can be used to characterise in more detail the contribtion of the endothelium to vascular health and disease and to identify and evaluate therapeutic targets for the treatment of vascular disease.

## Data Availability

Not applicable.
